# Multi-Elemental Analysis of Human Optic Chiasm—A New Perspective to Reveal the Pathomechanism of Nerve Fibers’ Degeneration

**DOI:** 10.3390/ijerph19074420

**Published:** 2022-04-06

**Authors:** Jacek Baj, Alicja Forma, Beata Kowalska, Grzegorz Teresiński, Grzegorz Buszewicz, Dariusz Majerek, Wojciech Flieger, Ryszard Maciejewski, Kaja Karakuła, Michał Flieger, Marcin Czeczelewski, Paweł Kędzierawski, Jolanta Flieger

**Affiliations:** 1 Chair and Department of Anatomy, Medical University of Lublin, 20-090 Lublin, Poland; wwoj24@wp.pl (W.F.); ryszard.maciejewski@umlub.pl (R.M.); pawelkedzierawski1@gmail.com (P.K.); 2 Chair and Department of Forensic Medicine, Medical University of Lublin, 20-090 Lublin, Poland; grzegorz.teresinski@umlub.pl (G.T.); g.buszewicz@umlub.pl (G.B.); michalflieeeger@gmail.com (M.F.); marcin.czeczelewski@gmail.com (M.C.); 3 Department of Water Supply and Wastewater Disposal, Lublin University of Technology, 20-618 Lublin, Poland; b.kowalska@pollub.pl; 4 Department of Applied Mathematics, University of Technology, 20-618 Lublin, Poland; d.majerek@pollub.pl; 5 Chair and I Department of Psychiatry, Psychotherapy, and Early Intervention, Medical University of Lublin, 20-439 Lublin, Poland; kaja.karakula@gmail.com; 6 Department of Analytical Chemistry, Medical University of Lublin, 20-093 Lublin, Poland

**Keywords:** trace elements, optic chiasm, optic nerve, alcohol use disorder (AUD), ICP-MS

## Abstract

The effect of metals on the functioning of the human eye is multifactorial and includes enzyme activity modulation, trace metal metabolic pathways changes, and cytotoxic activity. Functional dysfunctions appear mostly as a result of the accumulation of toxic xenobiotic metals or disturbances of micronutrients’ homeostasis. So far, the affinity of selected metals to eye tissues, i.e., the cornea, choroid, lens, and anterior chamber fluid, has been most studied. However, it is known that many eye symptoms are related to damage to the optic nerve. In order to fill this gap, the aim of the study is to perform a multi-element analysis of tissue collected postmortem from optic chiasm and optic nerves. A total of 178 samples from 107 subjects were tested. The concentrations of 51 elements were quantified by inductively coupled plasma mass spectrometry (ICP-MS) after the wet-mineralization step. In terms of elemental composition, the optic chiasm is dominated by two trace elements, i.e., iron (Fe) and zinc (Zn), besides macro-elements Ca, K, Na, P, and Mg. The subjects formed a homogeneous cluster (over 70% subjects) with the highest accumulation of aluminum (Al). The remaining two departing clusters were characterized by an increased content of most of the elements, including toxic elements such as bismuth (Bi), uranium (U), lead (Pb), chromium (Cr), and cadmium (Cd). Changes in elemental composition with age were analyzed statistically for the selected groups, i.e., females, males, and subjects with alcohol use disorder (AUD) and without AUD. A tendency of women to lose Se, Cu, Zn, Fe with age was observed, and a disturbed Ca/Mg, Na/K ratio in subjects with AUD. Although the observed trends were not statistically significant, they shed new light on the risks and possible pathologies associated with metal neurotoxicity in the visual tract.

## 1. Introduction

Metals are necessary for maintaining the health of the human body. Essential metals such as iron (Fe), zinc (Zn), copper (Cu), chromium (Cr), cobalt (Co), magnesium (Mg), manganese (Mn), nickel (Ni), and selenium (Se) possess many important physiological functions as, for example, enzyme cofactors, electron and oxygen transporters. Metals are also involved in protein modification, neurotransmitter synthesis, carbohydrate metabolism, immune response, redox reactions, etc. [1,2,3]. Essential metals’ deficiency may result in various neurological disorders, such as Fe deficiency, which causes restless legs syndrome, paroxysmal breath-hold, and in extreme cases, pediatric stroke, cranial nerve paralysis, or pseudotumor symptoms [4,5]. However, impaired neurotransmission and neurodegeneration can also result from damage caused by elevated levels of metals. The accumulation of essential or toxic metals leads to multidirectional intracellular damage, such as dysfunction of enzymes, mitochondria, DNA fragmentation, autophagy dysregulation, activation of apoptosis, incorrect protein folding, endoplasmic reticulum (ER) stress, or the generation of oxidative stress [6,7,8,9,10,11,12,13]. In the nervous system, these disorders are especially dangerous, leading to permanent injuries. Metals’ dyshomeostasis is associated with numerous neurodevelopmental diseases such as ADHD, autism spectrum disorder (ASD), epilepsy, neurodegenerative diseases such as Alzheimer’s disease (AD), Parkinson’s disease (PD), lateral atherosclerosis (ALS), and neurobehavioral deficits (migraines, major depressive disorders (MDD)) [14,15]. In addition, there is an association between elevated levels of certain metals and other neurological diseases such as Guillain–Barré disease (GBD), Gulf War Syndrome (GWS), Huntington’s disease (HD), manganism, multiple sclerosis, and Wilson’s disease (WD). Disturbed macro- and micro-element homeostasis has been observed in the brain of alcohol-dependent patients and even in those who died as a result of suicide [16,17,18,19,20].

According to the current state of knowledge, disturbances in the levels of micro- and macro-elements within the eye may be important in the pathogenesis of ophthalmic diseases, such as macular degeneration, diabetic retinopathy, glaucoma, or cataracts [21,22,23,24,25,26,27,28,29,30]. Most studies have been carried out on iron (Fe), zinc (Zn), copper (Cu), selenium (Se), and chromium (Cr). This choice is dictated by the participation of the above elements, presenting significant reduction potential, in the fight against oxidative stress caused by free oxygen radicals. An example is selenium (Se), which, being a key element of glutathione peroxidase, prevents oxidative damage within the lens and retina, similarly to zinc [27,28,29]. Elemental analysis of eye tissues can therefore be a useful tool for enabling the identification of the elemental deficiencies possibly underlying the dysfunctions of this organ. It is known that the balance of electrolytes is crucial for ocular homeostasis (i.e., lens transparency, retinal function, etc.), and that inorganic ions such as sodium, potassium, chloride, and calcium move through channels based on ion exchange (Na^+^/H^+^, Na^+^/Ca^2+^, HCO_3_^−^/Cl^−^) or active transport by the Na^+^/K^+^ ATPase [27]. The relationship between abnormal increases in sodium (Na) concentration or disturbed Na-K-ATPase activity and human lens opacity has been confirmed [11]. In turn, magnesium (Mg) deficiency, accompanied by ATPase impairment, may lead to the development of cataracts [29,30].

Optic neuropathies constitute a large group of ophthalmic disorders. Functional changes associated with damage to the optic nerve include decreased visual acuity, abnormal color vision (especially tritanomaly), impaired contrast sensitivity, and central or cecocentral scotomas [31]. The pathogenesis of optic neuropathy is not fully understood. The cause of the disease is related to inflammatory demyelination of the optic nerve. As patients overrepresent some human leukocyte antigens, genetic predisposition to optic neuritis seems to be of importance [32,33,34,35,36]. Studies have shown that chronic exposure to heavy metals may also contribute to optic neuropathy [37]. So far, cobalt (Co) neurotoxicity, leading to visual impairment and loss of vision, has been confirmed [38]. The toxic effect of lead on the optic nerve, retina, optic cortex, and lens has been confirmed [39]. Chronic exposure to mercury (Hg) [40,41,42] can cause abnormal color vision, narrowing of the visual field, poor night vision, or sudden double-sided blindness. Severe bilateral optic neuritis has been reported due to thallium (Tl) poisoning [43].

Most of the reported multi-elemental studies involve only some eye tissues such as the lens and anterior chamber fluid, which are usually collected during routine cataract surgery from living patients. However, the proper function of the visual pathway, which consists of the optic nerve that runs from the retina to the optic chiasm, is also responsible for proper vision. This segment of the visual pathway, made up of millions of nerve fibers, which are axons of the retinal ganglion cells, does not have specific characteristics of a peripheral nerve. In this study, for the first time, the ICP-MS elemental analysis of optic chiasm was described. The current study can therefore improve knowledge on the distribution of micro- and macro-elements in the visual tract and the identification of potential hazards. Since many metals are constituents of biomolecules important for normal eye physiology, this could shed light on the pathogenesis of many eye diseases.

As the human tissue elemental composition may indicate long-term disturbances in the electrolyte balance, the obtained results allow for answering the following questions: What kind of trends exist with age, gender, alcohol abuse in the optic chiasm, and optic nerve elemental composition? What kind of dangerous toxic elements influencing vision function undergo bioaccumulation in the optic chiasm? What kind of cross-inter-elemental interactions could be detected? To realize the above aims, a statistical analysis of experimental ICP-MS measurements involving 51 elements was conducted.

## 2. Materials and Methods

### 2.1. Studied Population

The tissues analyzed for the metal contents were collected in the Department of Forensic Medicine, University of Lublin, Lublin, Poland. The study was approved by the Local Ethical Committee (Medical University of Lublin, Poland, approval no KE-0254/217/2021) and the tissue collection was approved by the prosecutor’s office. The research was carried out in accordance with The Code of Ethics of the World Medical Association, Declaration of Helsinki, for experiments involving humans. The AUD group included the subjects with a medical history of chronic alcohol consumption (regardless of the alcohol concentration in the blood/urine/vitreous body confirmed by toxicological examination during the forensic medical dissection) while the no-AUD group included people without a history of an alcohol use disorder, which was confirmed by the medical documentation provided by the Prosecutor’s Office. Those deceased in advanced stages of decay were excluded from the study. The demographic characteristics of the groups are collected in Table 1. The study covers 107 subjects, and 78 of them had 2 independent samples taken, which gives us a total number of 187 measurements.

### 2.2. Sample Preparation

Wet mineralization of each 0.3–0.5 g sample (cut with a ceramic knife) was performed via the addition of 7 mL of 69% suprapur nitric acid HNO_3_ (Baker, Radnor, PA, USA), followed by heating to 190 °C in closed Teflon containers the microwave mineralization system TOPEX (PreeKem, Shanghai, China). After mineralization, 1 mL of HCl (Merck, Darmstadt, Germany) was added to stabilize some elements (As, Hg, Se, Mo, Tl, Ag). Finally, the samples were diluted to 25 mL by ultrapure water obtained in the purification system Milli-Q (Millipore, Darmstadt, Germany).

### 2.3. ICP-MS Measurements

The inductively coupled plasma mass spectrometer Agilent 8900 ICP-MS Triple Quad (Agilent, Santa Clara, CA, USA) was employed for elemental analysis. Most elements were analyzed in He mode (5.5 mL/min helium flow). Se and As were analyzed in O_2_ mode (gas O_2_ flow rate-30%). The plasma was working in general-purpose mode with 1.550 kW RF power, the nebulizer gas flow was 1.07 L/min, the auxiliary gas flow was 0.9 L/min, and Plasma gas flow was 15 L/min. Acquisition time was from 0.1 to 2 s, depending on the predicted concentration of the element. Due to the lack of certified reference material, the internal standard ISTD (Sc, Y, Lu) with a concentration of 0.5 ppm was used for the analysis. ISTD was added automatically using a standard mixing connector, the so-called mixing tee. The obtained recoveries were in the range of 80–120%. ICP commercial analytical standards were purchased from Agilent Technologies, Santa Clara, CA, USA (Multi-Element Calibration Standard 2A-Hg, Environmental Calibration Standard, Multi-Element Calibration Standard 2A), Merck Millipore, Darmstadt, Germany (ICP- Multi-Element Calibration Standard XVII, ICP- Multi-Element Calibration Standard VI, Phosphorus ICP standard), Honeywell Fluka™ analytical standards (Platinum Standard for ICP, Palladium Standard for ICP), and Inorganic Ventures, Christiansburg, Virginia, US (Rare Earth, Standards). The report, including background equivalent concentration-BEC, detection limit-DL, internal standard-ISTD, calibration equation, and the correlation coefficient R, together with individual calibration curves, is presented in Figure S1.

### 2.4. Statistics

In order to describe probability distributions of all elements, different measures of position and dispersion were used. Central tendency was described by mean and median. Minimum, maximum, standard deviation and quantiles of 25% and 75% were used to assess the dispersion. Relationships between particular elements were described by Spearman rank-order correlation coefficient. The choice of this coefficient was dictated by the strong asymmetry of the distribution of individual elements, and also the fact that the relationships were not always linear. Significances of correlation coefficients were determined by *t*-test [44].

Cluster analysis was performed to identify homogeneous groups of patients by concentration of elements in the body. This analysis was divided into two stages, the first using hierarchical cluster analysis using Ward’s method [45] to estimate the optimal number of clusters. In the second stage, the k-means method was applied with the number of clusters adopted from the hierarchical method [46]. The significance of differences between obtained clusters was determined by Kruskal–Wallis rank-sum test. A nonparametric test was used because of the abnormal distribution of analysed variables [47].

## 3. Results

### 3.1. Descriptive Statistics for ICP-MS Elemental Measurements

Two independent tissue samples from one patient were analyzed by ICP-MS for their composition, covering 51 elements. The difference between these measurements was tested with the paired-samples *t*-test. No element showed a necessity for rejecting the null hypothesis of the measurement equalities. Therefore, in each case, the arithmetic mean values of the measurements were analyzed. The paired *t*-test’s results prove the even distribution of elements in the nervous tissue. We can therefore conclude that the studied area is rather homogeneous in terms of elemental composition.

Descriptive statistics such as average concentrations of the individual elements, their range, median, and standard deviation (SD) are presented in Table 2. The highest concentration was achieved by elements such as P, Na, K, Ca, Mg in the order from the highest to the lowest. Their concentration was determined at a level exceeding 20 ppm. The group of the elements that had concentrations between 10 and 20 ppm includes Fe and Zn, whereas Rb, Cu, Al, and Sr achieved a concentration in the range of 0.5–10 ppm. In the range from 0.1 to 0.5 ppm, Ti, Se, Mn, and Ni are located. The remaining elements were quantified at a concentration below 0.1 ppm.

**Figure 1:**
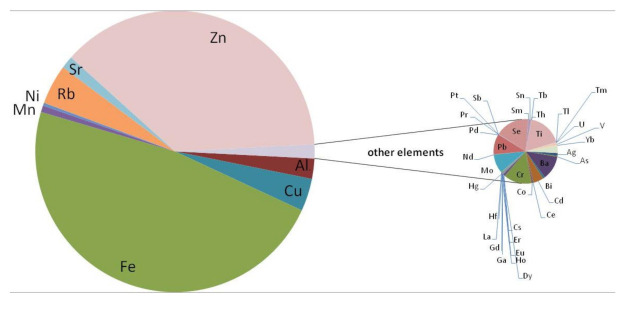
Pie of pie chart showing the percentage of trace elements (mean values) in the optic chiasm (*n* = 187). The series was split by a value of 0.110 ppm.

A pie chart was used to compare the proportions of elements and to visualize their percentages. Elements such as Ca, K, Na, P, and Mg, which are present in the highest concentration, were removed from the plot to show the proportions of trace elements in the examined tissue. As shown in Figure 1, iron (Fe) and zinc (Zn) are the most abundant trace elements in the optic chiasm. The obtained result confirms the presence of elements that are essential for the development and physiology of the eye. Unfortunately, in addition to macro- and micro-nutrients, xenobiotic metals are also absorbed and can accumulate in the visual tract, leading to structural and physiological damage. Particular attention is paid to the high average content of Al.

**Figure 2:**
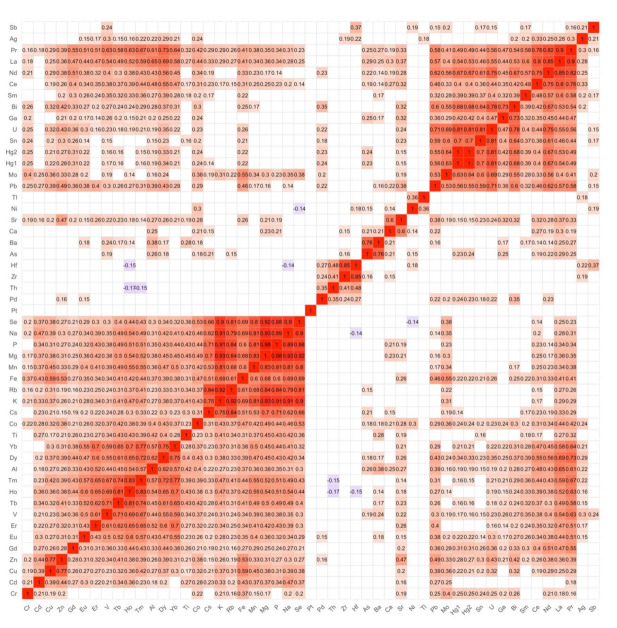
Spearman rank-order correlation matrix for studied population.

The correlation matrix (Figure 2), prepared for the collected data, reveals many positive correlations of different strengths, expressed by the Spearman rank-order correlation coefficient. The correlations are rather weak, with the Spearman’s r smaller than 0.5. There are seven main clusters, which, due to their size, can be arranged as follows: (Pb, Mo, Hg, Sn, U, Ga, Bi, Sm, Ce, Nd, La, Pr); (Yb, Dy, Al, Tm, Ho, Tb, V, Er, Eu); (Se, Na, P, Mg, Mn, Fe, Rb, K); (Hf, Zr, Th, Pd); (Zn, Cu, Cd); (Ba, As); (Sr, Ca). The inter-element correlations of greater importance with the Spearman’s r greater than 0.8 concern the correlations of Se with Na, K, Rb, Mg, Mn, P; Na with K, Mg, Mn, P; K with Rb, Mg, Mn, P; Mg with P, Mn, Rb, and Cs with Rb; P with Rb, Mg; La with Nd, Ce; Pr with La, Nd; U with Sn, Hg; Zr with Hf.

### 3.2. Cluster Analysis

**Figure 3:**
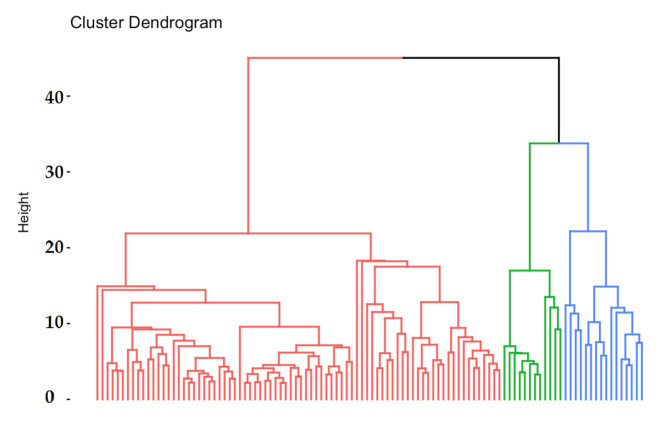
The results of Ward hierarchical cluster analysis. Based on distance (vertical axis), three clusters were chosen. The numbers on the X-axis represent the observation numbers.

**Figure 4:**
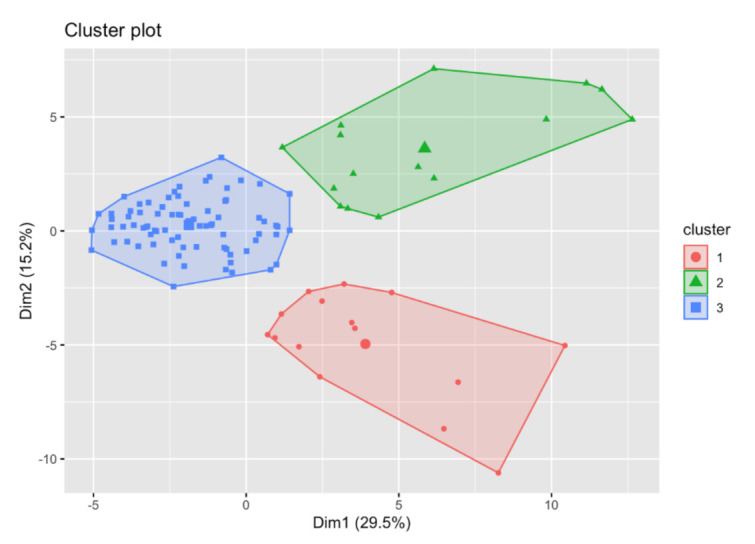
Results of cluster analysis mapped on 2D PCA surface. Points represent observations of three clusters with linear combination envelopes.

Based on the hierarchical cluster analysis, the potential number of clusters was assessed. Then, using the k-means method, the division into the optimal number of clusters was made. Principal Component Analysis (PCA) was used to illustrate the results. In order to represent potential clusters in 2- or 3-dimensional space, the dimensions have been reduced. From the original number of elements, three of them, i.e., Th, and Pd, were excluded as they exhibited no variation. There were 49 elements left for further analysis. The assessment of the grouping potential of objects in the data set was made on the basis of the distance matrix and the Ward hierarchical method (Figure 3). The division into three clusters using the k-means method presented in the space of the first two main components is shown in Figure 4. One can clearly see the separation of observations between the clusters.

Descriptive statistics of individual elements with a division into clusters are presented in Table 3. Most of these differences are statistically significant.

The obtained grouping of cases shows a relatively homogeneous distribution of the studied population in terms of the content of elements. Cluster 3, marked in blue in Figure 4, has the highest number of subjects, i.e., 78, while the other two have 15 subjects each. The content of elements is usually lower in the most numerous cluster than in the remaining ones. Noteworthy is the high content of Al, the average concentration of which is several hundred times higher than in the other two. In clusters 1 and 2, the content of most elements is significantly higher than in cluster 3. For example, in cluster 1 we observe several times higher concentrations of Pb, Sb, Sn, U and Bi, while in cluster 2, apart from macro-elements such as Na, K, Mg, Se, and toxic metals such as Cd, Cr and Mn are present in the highest concentrations.

### 3.3. Age, Gender, and Alcohol Abuse Influence on the Elemental Composition

**Figure 5:**
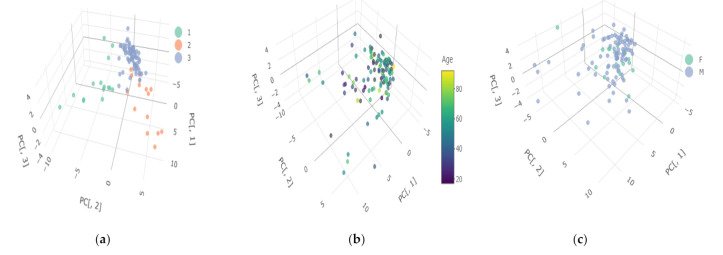
Selected clusters covering all examined subjects (*n* = 187) in 3D space: (**a**) 1st cluster—red, 2nd cluster—green; 3rd cluster—blue; (**b**) examined subjects colored depending on the age; (**c**) examined subjects colored depending on the gender according to the attached legends.

The performed cluster analysis allows the study population to be divided into three homogeneous clusters. It should be emphasized that most of the cases, i.e., over 70%, are located in one cluster (blue cluster 3). As can be seen in Figure 5, no clear relationship can be found between age, gender, and membership in clusters. Taking into account the division into a control group and a test group, including people with chronic alcohol abuse also did not reveal any clear structure of the division. Clusters (1—red, 2—green) are characterized by a much smaller number of members and a significantly higher content of elements compared to cluster 3. The reason for the separation of these clusters is unknown and may have its source in the diet or living environment.

A comparative analysis of the concentration of individual elements depending on both age and whether the person was addicted to alcohol or not did not show clear relationships. In most cases, horizontal regression lines were obtained that would suggest no relationship between age and element concentration at the optic chiasm. However, in some cases, the obtained relationships are interesting. For example, for Tb in the group without AUD, there is no relationship between age and concentration, but in the AUD group, the relationship is positive. There are also opposite situations, for example for Sm in the group without AUD, there is no relationship between age and concentration, and in the group addicted to alcohol, Sm concentration decreases with age. Interpretation of some relationships is hampered by outliers (as in the case of Pt).

**Figure 6:**
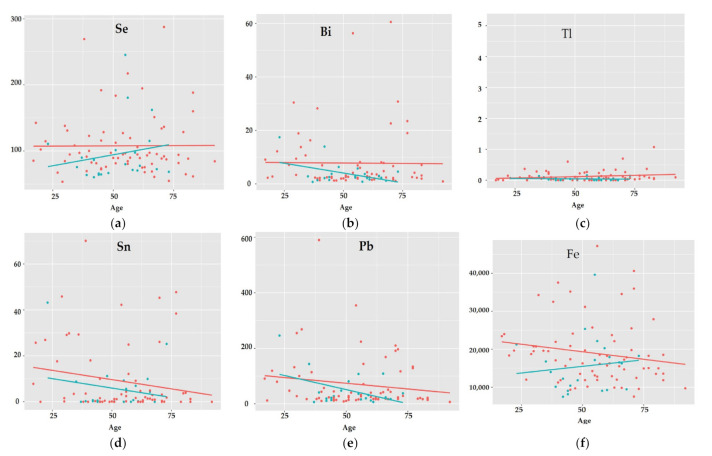
The trends of changes occurring in the concentration of elements (ppb): Se (**a**), Bi (**b**), Tl (**c**), Sn (**d**), Pb (**e**), Fe (**f**) in the optic chiasm with age for the control group—without AUD (red points, red line) and the test group with AUD (green points, green line).

Most cases involved a situation where in the group without AUD, but with age, no clear changes in the concentration of elements at the optic junctions were observed, but in the AUD group, there was an increase in the concentrations of Cd, Co, Ti, V, Zr, K, Mg, Na, Ni, Se, and Tb (Figure 6a), or a decrease in Ag, Al, As, Bi, Ca, Ce, Cs, Eu, La, Nd, Sm, Sr (Figure 6b).

For both AUD and without-AUD groups, changes in the concentration of elements with the age of the patients followed the same course, i.e., no visible changes for Ga, Gd, Hf, Tl, Tm, Yb, Zn, P, Pt, Rb, or Sb (Figure 6c), or concentrations decreased with age in both groups in a parallel way for Dy and Sn (Figure 6d), or faster in cases of alcohol abusers for a few other elements, such as Hg, Pb, Pr (Figure 6e).

An interesting group of elements is those ones whose content in the optic chiasm decreases with age in the group without AUD, but increases for the AUD group. This was the case for Cr, Cu, Er, Fe, Ho, V, and Mn (Figure 6f).

Only individual cases concerned a situation where an increase was observed in the group without AUD, while for the AUD group, there was a decrease in concentration (Ba), or where in the group without AUD the concentration decreased with age, and in the AUD group there was no change (Mo).

**Figure 7:**
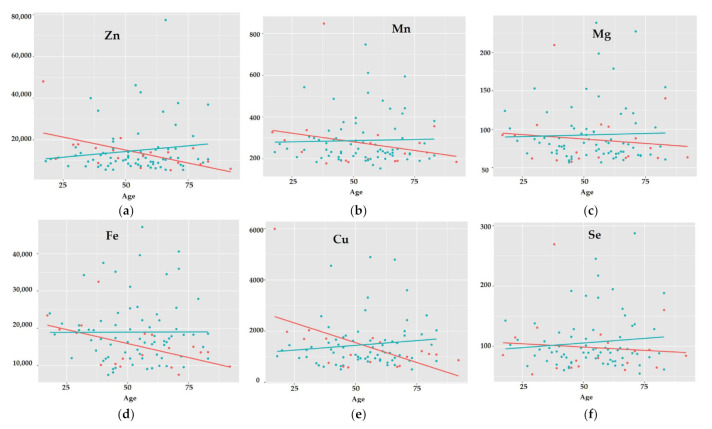
The trends of changes occurring in the concentration of elements (ppb): Zn (**a**), Mn (**b**), Mg (**c**), Fe (**d**), Cu (**e**), Se (**f**) in the optic chiasm with age for males (green points, green line) and females (red points, red line).

Regarding the influence of gender on the observed trends and changes in the elements’ concentrations of the optic chiasm while considering age (Figure 7), the following was observed: (i) there is no change or a slight increase in element concentrations for Zr, Cr, Er, Gd, Hf, Ni, P, Pt, Sb, Tb, and Ti with age for both females and males; (ii) a parallel decrease with age for females and males in the concentrations of Hg, U, Sn, Pr, Pb, Mo; (iii) no changes in men and an increase in concentration with age for women for Nd and Tl, or a decrease in concentration in women for Mn, Mg, La, K, Ho, Fe, Eu, Dy, Cu, Cs, Nd, V, and Yb; (iv) females lose Tm, Sr, Sm, Se, Rb, Na, Ga, Zn, Co and Na in the optic chiasm with age as opposed to males.

**Figure 8:**
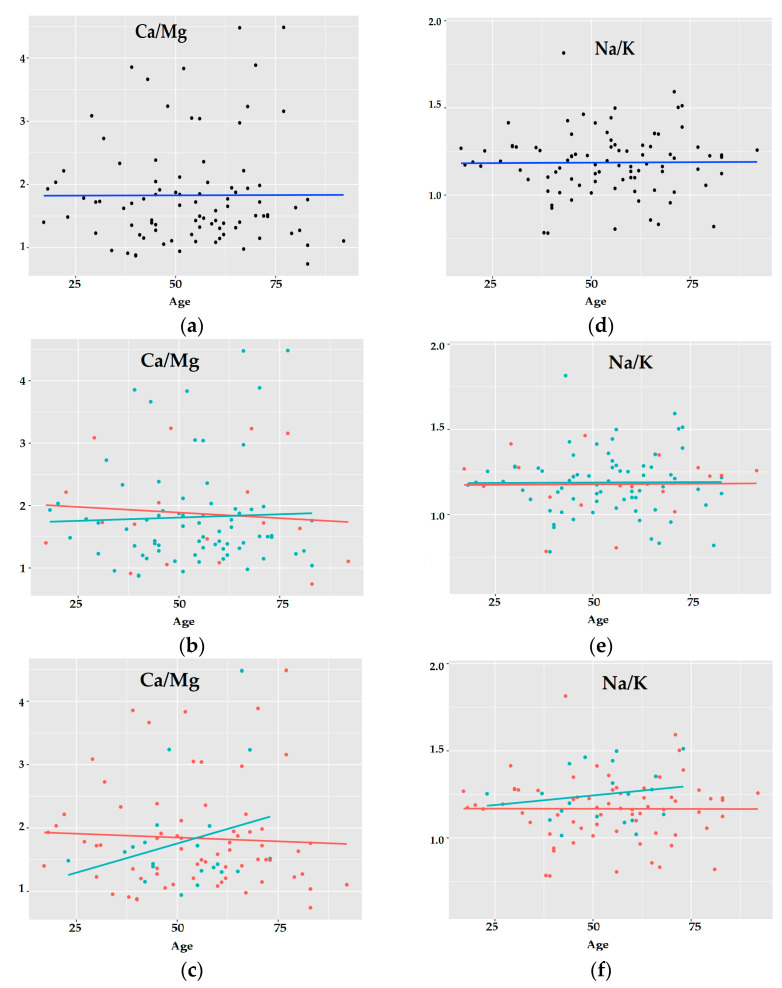
The trends of changes for Ca/Mg and Na/K ratios with age for all 187 subjects (**a**,**d**) (back points, blue line); males (green points, green line) and females (red points, red line) (**b**,**e**); AUD subjects (green points, green line) and the subjects without AUD (red points, red line) (**c**,**f**).

Taking into account all the subjects, there is no visible effect of age on the concentration of elements such as Ca, Mg, K, or Na, nor the Ca/Mg, Na/K ratios in the optic chiasm (Figure 8a,d). After taking gender into account, it can be seen that the ratio of Ca/Mg in women slightly decreases, while in men it is at an almost constant level (Figure 8b,e). The breakdown between alcohol abusers and subjects without AUD also showed (even greater) differences compared to age and gender. In the AUD group, the growing tendencies concerning the changes in the Ca/Mg and Na/K ratios can be noticed, in contrast to subjects without AUD (Figure 8c,f).

## 4. Discussion

Multiple-element quantification by the ICP-MS method showed high levels of Fe and Zn in the tissue of optic chiasm. The dominant content of these trace elements is justified, as Fe is essential for the physiological development of the eye, and its deficiency at early ages can cause dysfunctions such as angiodysplasia, changes in eye movement, and even blindness. Reduction in systemic iron levels as a consequence of selected medication intake (desferroxamine), medical procedures (transfusions), or diseases (Eales’ disease), can also cause disturbances in electrophysiological status that result in blurred vision [48,49]. Without a doubt, Fe is necessary for the proper functioning of the eye, but iron overload is toxic and leads to the production of oxygen free radicals, which are the source of protein, lipid and DNA damage in the Fenton reaction. The Fe overload state is believed to be involved in the pathogenesis of age-related macular degeneration (AMD). Ocular histopathology confirmed that the retinas of patients with AMD contain more iron than the retinas of healthy people. However, it should be emphasized that more research is needed to establish the relationship between disturbed iron homeostasis and AMD [50,51].

In turn, Zn is the second most abundant trace element in the human body. However, the Zn transport mechanism is poorly understood. So far, several proteins involved in zinc transport have been described [52]. It is known that plasma zinc binds with protein and a2-macroglobulin, partially with low-molecular-weight amino acids (histidine and cysteine), and intracellularly with metallothioneins [53,54].

Zn plays a fundamental role in cellular metabolism, where it exercises catalytic and structural functions as a component of many metalloenzymes and transcription factors. Moreover, Zn is involved in maintaining the function of the cell membrane [55]. It turns out that the concentration of Zn in eye tissues is higher than other tissues [56]. Zn concentration has been quantified so far in the retina and choroid, ciliary body, iris, optic nerve, sclera, cornea and lens in animal tissues, i.e., rat and cattle photoreceptors, cat and dog tapetum lucidum, and fish [56]. In human tissues, the highest concentrations of Zn were found in the choroid and retina. The importance of Zn for proper vision may be demonstrated by the symptoms of Acrodermatitis enteropathica, which is a rare disease caused by an insertion mutation affecting the mRNA of the zinc transport protein [57]. This disease includes many ocular symptoms such as blepharitis, conjunctivitis, cataracts, surface point opacities, corneal opacities, nebular subepithelial opacities, photophobia, and linear corneal erosions [56]. All the symptoms of Zn deficiency confirm its role in the function of normal vision. Deficiencies caused by impaired absorption of Zn in Crohn’s disease, in patients with parenteral nutrition, and in alcoholism and liver cirrhosis, cause visual disturbances, retinitis pigmentosa, cherry red maculopathy, and impaired dark adaptation [58,59,60]. It is commonly believed that supplementation with Zn also slows the progression of AMD. However, there is no general consensus on this issue. Randomized controlled trials (RCTs), prospective cohort studies, retrospective cohort studies, and case–control studies published in 2013 are inconclusive. It has only been shown that Zn supplementation may be effective in preventing progression to advanced AMD, but is not sufficient to induce clinically significant changes in visual acuity [61]. According to studies by Blasiak et al. [62], the effect of Zn in AMD may be modulated by the substrate genetically, and the controversy may be explained by the zinc–autophagy–AMD triad.

Descriptive statistics made on the basis of ICP-MS measurements indicate, apart from the dominant amounts of Fe and Zn, a high level of Al at the optic chiasm. The high content of this element characterizes all the objects grouped in the largest cluster of over 70% of the analyzed samples, which proves the ability of Al to bioaccumulate in the nervous tissue. Al is a metal that occurs naturally in many industries. Al is a vaccine adjuvant and a component of drugs, including antacids, and is in food additives and cosmetics. Therefore Al can occur as an impurity in many products. It should be emphasized, however, that the main source of Al is food (>90%) for the general public. Al is absorbed both through the digestive system and through the respiratory system and even through the skin. At this stage, the bioavailability of Al is enhanced by the acidic environment, and the absorption restriction is provided by silicon-containing compounds. In the human body, Al ions have an affinity mainly toward negatively charged, oxygen-donor ligands, such as carboxylate and phosphate groups located in proteins, RNA, DNA, amino acids, nucleotides, citrate, phytate, lactate, carbonate, phosphate, and sulfate [63]. In the serum, Al^3+^ ions bind to the trivalent metal ion carrier, i.e., the Tf protein in the form of a citrate complex. Al has been shown to be particularly dangerous for the functioning of the central nervous system, causing cognitive and neurodegenerative disorders. Many studies have shown that exposure to Al can cause neuropsychiatric and neurological symptoms, as well as diseases such as Alzheimer’s disease, risk of cognitive impairment, dementia, allergic conditions, inflammatory skin lesions, and a higher risk of developing lung cancer or bladder cancer [64,65,66,67]. Yoshimasu et al. found higher concentrations of Al in the brain of victims of amyotrophic lateral sclerosis (ALS) [67]. Particularly dangerous is the inhalation of aluminum compounds in the form of nanoparticles, which are more toxic compared to larger micro-sized particles [68]. Al toxicity is a result of damage to the mitochondria and the integrity of the mitochondrial membrane. Al-induced damages are possibly mediated by the induction of the caspase-3 gene [69]. It is known that the functioning of nervous networks requires efficient neurotransmission, and the presence of Al in the CNS causes changes in electrical excitability, impairs synaptic transmission, and voltage-controlled ion channels [70]. Specifically, Al^3+^ inhibits voltage-gated Ca^2+^ channels by reducing the maximal velocity of Ca^2+^ influx and inhibiting neurotransmitter receptors [71]. Studies have shown that long-term exposure to the Al environment could increase the activity of the glycogen synthase kinase-3ß (GSK-3ß) and then inhibit signal transduction [72]. Fry et al. suggested that Al-induced neurofibrillary degeneration had similarities to Alzheimer-related changes [73]. Other work has substantiated that Al does locate in neurological tissues and can inhibit the vestibule–ocular reflex [74].

In turn, clusters 1 and 2 are distinguished by increased concentrations of most elements, including very toxic elements that may affect processes related to vision. For example, cluster 1 is characterized by an increased level of Ga (four times compared to cluster 3), Pb (five times compared to cluster 3), and U (ten times compared to cluster 3). Pb accumulation is known to affect the optic nerve, retina, optic cortex, lens, and extra- and intraocular muscles. The effects of chronic Pb exposure may be a reduction in the retinal nerve fiber layer, macular and choroidal thickness [75]. As early as in 1968, Baghdassarian described a case of bilateral optic neuropathy due to systemic Pb poisoning [76]. Contemporary studies confirm this observation. In 2019, Abri Aghdam et al. reported the case of a patient suffering from painless Pb-induced optic neuropathy [39]. In 2005, Patel and Athawale prepared a case-study of an 11-month-old child with acute encephalopathy as a result of Pb poisoning resulting in optic neuropathy [77].

Additionally, the remaining metals, i.e., Ga and U, which were detected in increased amounts in cluster 1, may cause ocular symptoms in the case of chronic exposure. Cluster 1 shows a 10-fold higher content of U compared to the dominant cluster 3. U is known to be a potent carcinogen, but little is known about its ocular toxicity. Animal tests reveal the effect of systematic uranium intoxication on cataract formation and optic atrophy [78]. Ga bioaccumulation is also harmful as it can cause optic neuropathy by interacting with transferrin receptors, and consequently, disturbing the function of optic nerve oligodendrocytes [79]. In cluster 2, the increased content of Cd and Cr is noticeable. Xenobiotic ions such as Cd, along with Pb, are the main causes of metal-induced ophthalmic toxicity in occupational exposure [80]. It is known that Cd can accumulate in epithelial tissues, lens and cornea. The changes it induces are irreversible and are of the nature of mitochondrial edema [81]. In addition to its direct cytotoxic effect on cells, Cd has a competitive effect on the trace metal pathways, disturbing the balance of Ca, Cu and Fe.

Changes in the concentration of elements in the tissue of the optic chiasm occurring with age in women and men as well as in the group with and without AUD seem interesting. While gender, age, or chronic alcohol abuse do not affect cluster formation, certain trends of changes occurring with age in the selected groups can be observed. For subjects in the AUD group, the levels of Se and Fe increase with age, and the levels of toxic elements such as Bi and Pb decrease with age, which paradoxically seems to be beneficial. With age, women are exposed to the loss of valuable elements such as Fe, Mg, Cu, Se, and Zn. In the AUD group, there was a clear upward trend in the Ca/Mg and Na/K ratios, while in the non-AUD group, these ratios remained constant with age. This is further evidence that alcoholism causes the key electrolyte imbalances that disrupt the maintenance of basic bodily functions, such as nerve conduction.

## 5. Conclusions

Functional changes associated with damage to the optic nerve reveal many clinical symptoms including decreased visual acuity, abnormal color vision (especially tritanomaly), impaired contrast sensitivity, and central or cecocentral scotomas. Xenobiotic metals, as neurotoxins, may take part in this damage. On the basis of the performed tests, a dangerous affinity of the optic nerve tissue was found, first of all, to Al, but in exceptional cases also Pb, U, Bi, Cr, and Cd. Factors such as age, gender or alcohol abuse did not affect the bioaccumulation of these metals. The observed trends of changes in the content of macro- and micro-nutrients occurring with age show the tendency of women to lose important elements such as Se, Cu, Zn, and Fe and a disturbed balance of key elements in nerve signaling (Ca/Mg, Na/K) in individuals chronically abusing alcohol.

## Figures and Tables

**Table 1:** The demographic characteristic of the groups enrolled in the study.

Population	Gender	Groups	*n*	%	Min-Max Age	Median Age	Mean Age ± SD
*n* = 107	Female *n* = 20	AUD	5	4.67	39–68	46.5	50.0 ± 12.5
		no-AUD	15	14.02	17–92	57.0	55.1 ± 23.6
	Male *n* = 87	AUD	20	18.69	23–73	55.0	51.4 ± 12.7
		no-AUD	67	62.62	18–83	55.0	54.6 ± 16.2

**Table 2:** Descriptive statistics for ICP-MS measurements of optic chiasm samples taken from the entire population of the studied subjects (*n* = 187).

Element	*n*	Min	Max	Median	q1	q3	Mean	sd
Ag ^1^	187	0.000	63.479	5.096	1.974	12.170	8.388	9.751
Al ^1^	187	53.624	5927.694	589.058	311.609	1054.391	849.046	853.492
As ^1^	187	0.000	13.089	2.032	0.980	3.560	2.828	2.689
Ba ^1^	187	9.969	632.037	37.049	24.635	63.394	70.656	92.218
Bi ^1^	187	0.000	0.000	0.000	0.000	0.000	0.000	0.000
Ca ^2^	187	0.458	119.576	2.662	1.716	4.613	6.055	11.351
Cd ^1^	187	56.127	10,013.799	128.430	94.403	191.961	210.129	728.266
Ce ^1^	187	1.861	182.218	22.242	12.818	39.591	30.777	28.842
Co ^1^	187	0.399	19.346	1.765	1.139	3.189	2.617	2.538
Cr ^1^	187	0.716	15.915	2.434	1.869	3.206	2.888	1.900
Cs ^1^	187	0.000	1183.577	26.712	5.564	109.331	83.472	145.685
Cu ^1^	187	1.219	21.430	5.289	4.082	7.437	6.353	3.762
Dy ^1^	187	354.078	11,196.279	1123.961	734.256	1603.526	1359.101	1133.862
Er ^1^	187	0.000	2.230	0.483	0.312	0.677	0.542	0.347
Eu ^1^	187	0.172	6.313	1.035	0.771	1.392	1.208	0.740
Fe ^1^	187	0.070	1.253	0.359	0.244	0.489	0.384	0.201
Ga ^1^	187	6401.308	59,353.704	14,982.996	10,850.651	20,567.069	17,299.171	9122.571
Gd ^1^	187	0.000	16.935	0.169	0.000	0.875	0.585	1.386
Hf ^1^	187	0.070	4.699	0.638	0.447	0.949	0.799	0.646
Hg1 ^1^	187	0.000	10.691	0.000	0.000	0.000	0.507	1.555
Hg2 ^1^	187	0.000	24.400	0.070	0.000	2.905	2.213	4.119
Ho ^1^	187	0.000	24.184	0.122	0.000	2.925	2.219	4.134
K ^2^	187	0.000	2.006	0.601	0.476	0.802	0.671	0.296
La ^1^	187	475.689	6120.696	1645.623	1400.857	2061.010	1886.702	906.945
Mg ^2^	187	0.527	8.063	1.278	0.995	1.977	1.664	1.127
Mn ^1^	187	48.281	304.736	73.983	66.302	92.716	88.194	39.955
Mo ^1^	187	122.010	858.483	232.228	200.294	288.796	273.634	124.911
Na ^2^	187	0.000	261.006	38.232	9.743	84.503	56.004	57.347
Nd ^1^	187	743.445	7668.469	1914.792	1628.342	2329.664	2207.223	1075.248
Ni ^1^	187	0.000	5.992	0.561	0.279	1.077	0.915	1.031
P ^2^	187	0.000	3744.519	0.000	0.000	58.293	121.754	401.851
Pb ^1^	187	1533.439	9474.153	2489.203	2151.460	3001.225	2874.485	1336.601
Pd ^1^	187	4.896	590.073	23.203	15.087	50.737	59.341	95.678
Pr ^1^	187	0.000	1.207	0.000	0.000	0.000	0.012	0.102
Pt ^1^	187	0.081	2.431	0.478	0.312	0.687	0.558	0.353
Rb ^1^	187	0.000	14.589	0.050	0.026	0.092	0.219	1.304
Sb ^1^	187	348.990	6396.930	1466.236	1132.742	1854.360	1666.120	877.629
Se ^1^	187	0.000	67.807	0.000	0.000	0.000	1.171	6.615
Sm ^1^	187	46.901	371.195	87.448	73.823	109.518	100.926	47.430
Sn ^1^	187	0.000	1.532	0.254	0.053	0.529	0.334	0.322
Sr ^1^	187	0.000	119.631	0.887	0.000	4.333	8.101	17.876
Tb ^1^	187	74.626	4117.310	363.803	228.190	651.124	521.521	488.893
Th ^1^	187	0.088	1.324	0.415	0.305	0.557	0.456	0.212
Ti ^1^	187	0.000	0.355	0.000	0.000	0.000	0.002	0.026
Tl ^1^	187	24.151	617.923	73.064	52.070	104.722	102.144	101.168
Tm ^1^	187	0.000	9.376	0.045	0.000	0.118	0.152	0.701
U ^1^	187	0.031	1.871	0.473	0.367	0.638	0.523	0.242
V ^1^	187	0.000	17.502	0.421	0.224	0.800	1.456	2.977
Yb ^1^	187	2.613	21.271	6.103	4.693	7.987	6.845	3.212
Zr ^1^	187	0.000	3.078	0.561	0.384	0.790	0.639	0.405
Zn ^1^	187	4302.780	89,710.452	10,289.986	7117.469	14,991.362	13,633.378	11,779.637

^1^ measured in ppb (ng/g wet weight); ^2^ measured in ppm (µg/g wet weight).

**Table 3:** The elements’ mean, (SD) values in ppb with a significance of division into clusters (*p*-value) calculated using the Kruskal–Wallis rank-sum test.

Element	1st Cluster	2nd Cluster	3rd Cluster	*p*-Value
Ag	12, (10)	6, (7)	8, (9)	0.12
Al	1211, (510)	1376, (1475)	695, (467)	<0.001
As	3.54, (2.30)	3.15, (2.41)	2.60, (2.25)	0.067
Ba	101, (72)	74, (94)	61, (71)	<0.001
Bi	22, (17)	5, (3)	4, (4)	<0.001
Ca ^1^	537, (1249)	238, (110)	135, (58)	<0.001
Cd	31, (18)	67, (49)	25, (15)	<0.001
Ce	5.34, (2.13)	3.23, (1.58)	2.02, (1.38)	<0.001
Co	3.66, (0.89)	4.18, (1.61)	2.51, (1.21)	<0.001
Cr	160, (98)	186, (290)	62, (99)	<0.001
Cs	6.2, (4.0)	11.0, (4.4)	5.8, (2.6)	<0.001
Cu	1723, (563)	2439, (1434)	1166, (707)	<0.001
Dy	0.72, (0.24)	0.83, (0.30)	0.46, (0.19)	<0.001
Er	1.34, (0.37)	1.80, (0.59)	1.06, (0.50)	<0.001
Eu	0.50, (0.17)	0.52, (0.21)	0.34, (0.14)	<0.001
Fe	20,356, (5297)	29,730, (9682)	15,266, (6173)	<0.001
Ga	1.62, (2.16)	0.39, (0.59)	0.43, (0.50)	0.006
Gd	1.33, (0.57)	1.19, (1.02)	0.66, (0.50)	<0.001
Hf	0.99, (1.69)	0.08, (0.29)	0.47, (1.34)	0.023
Hg1	8.5, (3.5)	2.2, (3.3)	1.2, (1.8)	<0.001
Hg2	8.53, (3.46)	2.10, (3.25)	1.20, (1.81)	<0.001
Ho	0.77, (0.17)	1.02, (0.26)	0.59, (0.17)	<0.001
K ^1^	1672, (465)	3627, (1130)	1698, (457)	<0.001
La	3.08, (0.97)	2.15, (0.74)	1.31, (0.49)	<0.001
Mg ^1^	84, (21)	165, (48)	78, (17)	<0.001
Mn	269, (65)	511, (162)	241, (60)	<0.001
Mo	129, (40)	92, (54)	40, (31)	<0.001
Na ^1^	1987, (455)	4301, (1322)	1958, (487)	<0.001
Nd	2.58, (0.90)	1.03, (0.66)	0.60, (0.38)	<0.001
Ni	174, (200)	43, (114)	121, (376)	<0.001
P ^1^	2657, (761)	5394, (1600)	2542, (574)	<0.001
Pb	196, (133)	67, (68)	37, (41)	<0.001
Pr	0.98, (0.29)	0.73, (0.23)	0.44, (0.17)	<0.001
Pt	0.09, (0.05)	0.12, (0.10)	0.24, (1.41)	<0.001
Rb	1411, (596)	3143, (1191)	1523, (516)	<0.001
Sb	5.19, (12.73)	0.10, (0.36)	0.54, (1.94)	<0.001
Se	93, (23)	191, (64)	90, (23)	<0.001
Sm	0.62, (0.23)	0.35, (0.26)	0.27, (0.20)	<0.001
Sn	36, (15)	3, (4)	4, (9)	<0.001
Sr	813, (480)	688, (343)	452, (348)	<0.001
Tb	0.51, (0.10)	0.69, (0.18)	0.40, (0.13)	<0.001
Ti	93, (34)	198, (129)	87, (64)	<0.001
Tl	0.17, (0.18)	0.11, (0.14)	0.16, (0.59)	0.043
Tm	0.58, (0.18)	0.77, (0.25)	0.47, (0.14)	<0.001
U	6.61, (3.50)	0.84, (0.59)	0.67, (1.02)	<0.001
V	8.18, (2.66)	9.11, (3.03)	6.00, (2.23)	<0.001
Yb	0.78, (0.23)	1.04, (0.44)	0.54, (0.22)	<0.001
Zn	21,318, (10,884)	23,546, (12,668)	11,058, (8307)	<0.001
Zr	31, (33)	12, (14)	22, (34)	0.14

^1^ measured in ppm (µg/g wet weight).

## Data Availability

Data supporting reported results can be presented by A. Forma on request.

## Supplementary Materials

The following supporting information can be downloaded at: https://www.mdpi.com/article/10.3390/ijerph19074420/s1, Figure S1: background equivalent concentration-BEC, detection limit-DL, internal standard-ISTD, calibration equation, and the correlation coefficient R, together with individual calibration curves report.
