# Supplementary figures and images for: Multi-Elemental Analysis of Human Optic Chiasm—A New Perspective to Reveal the Pathomechanism of Nerve Fibers’ Degeneration
Source: Int J Environ Res Public Health. 2022 Apr 6;19(7):4420. doi: 10.3390/ijerph19074420 (PMC8998695; doi:10.3390/ijerph19074420)

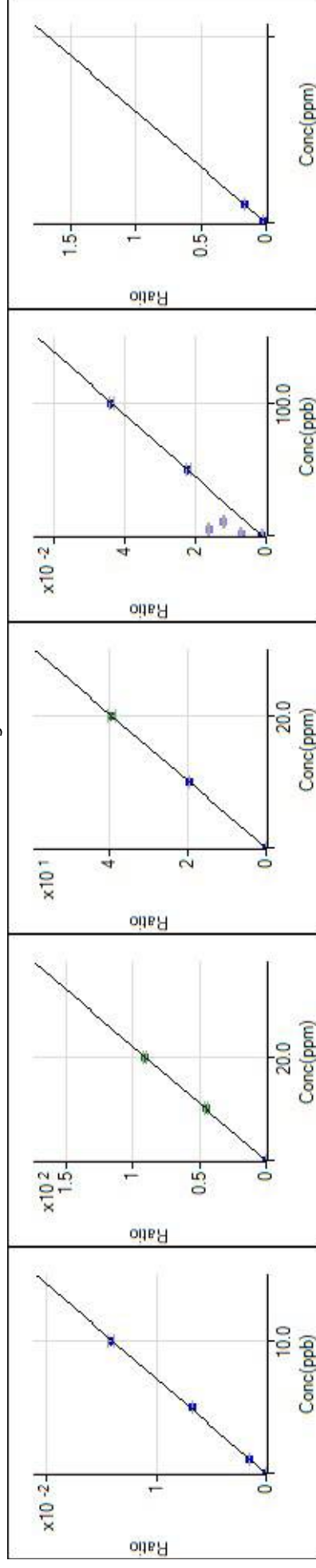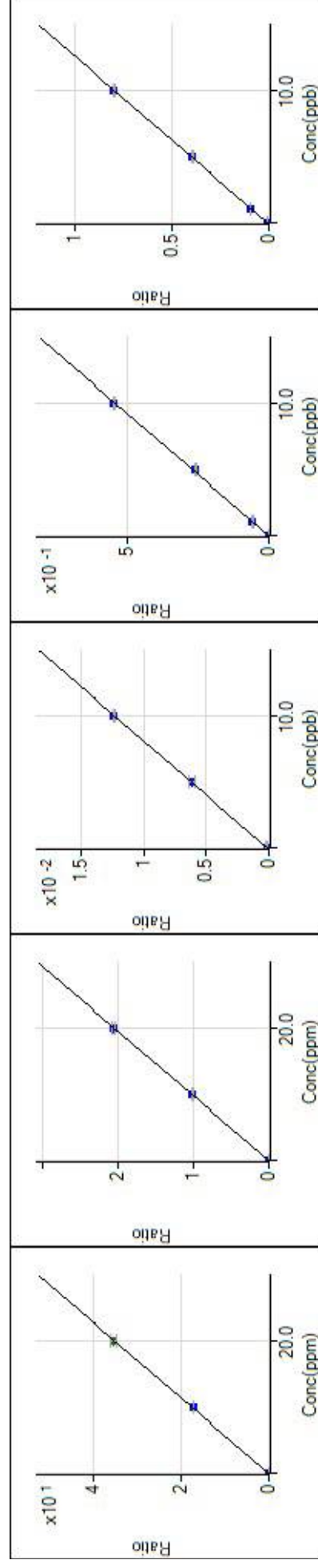

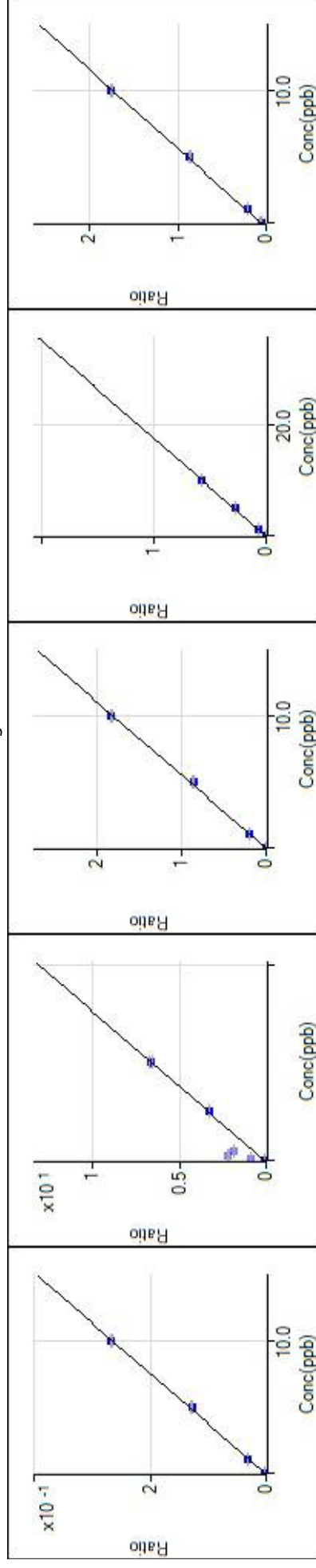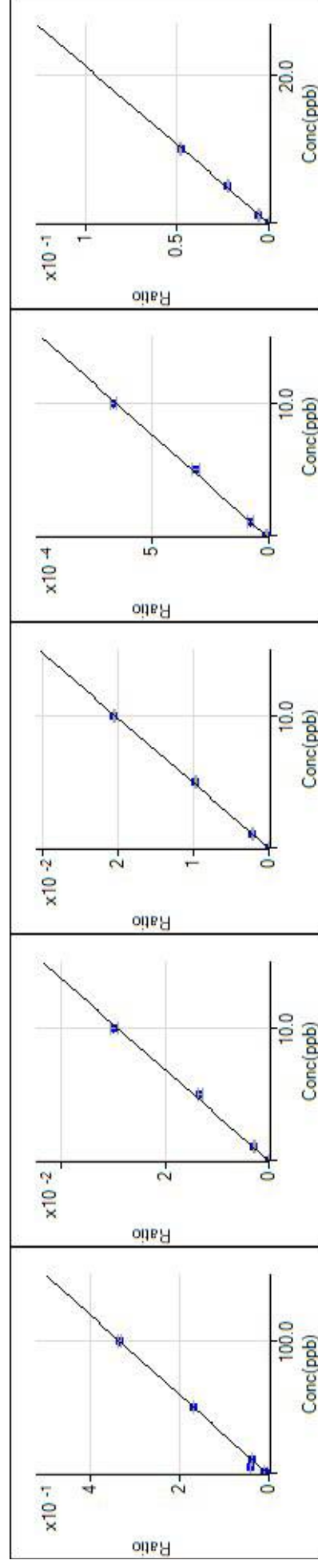

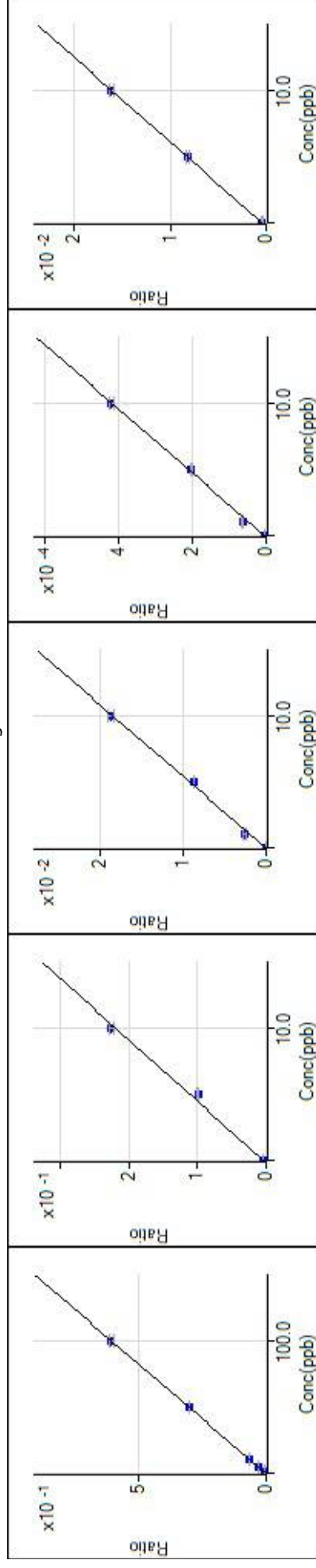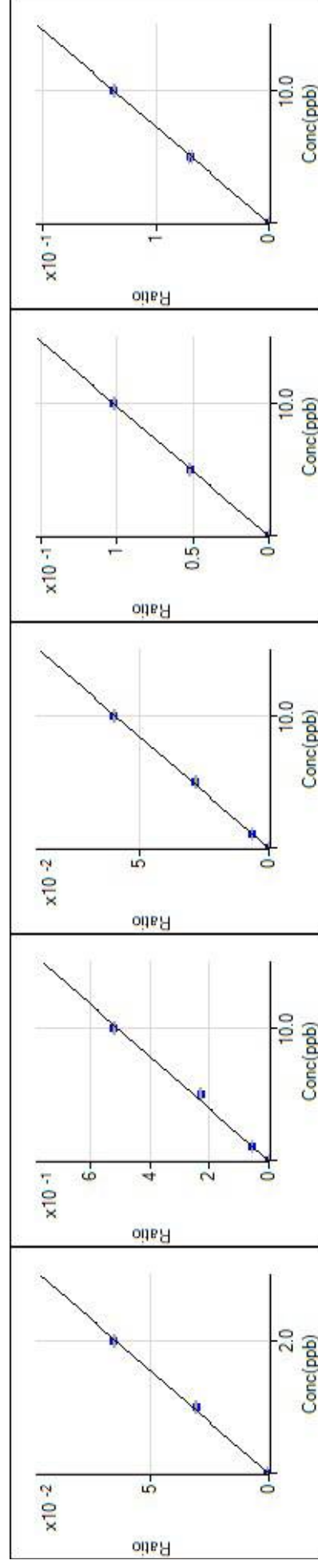

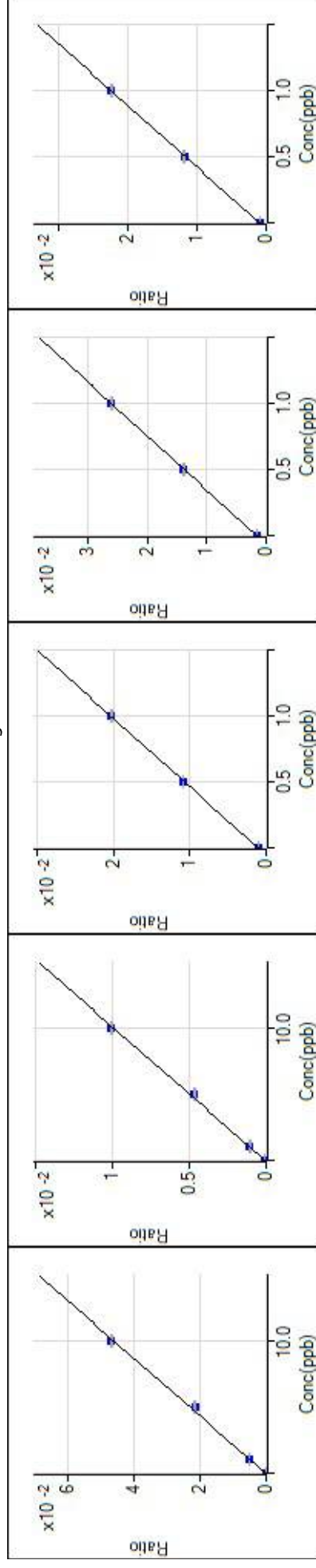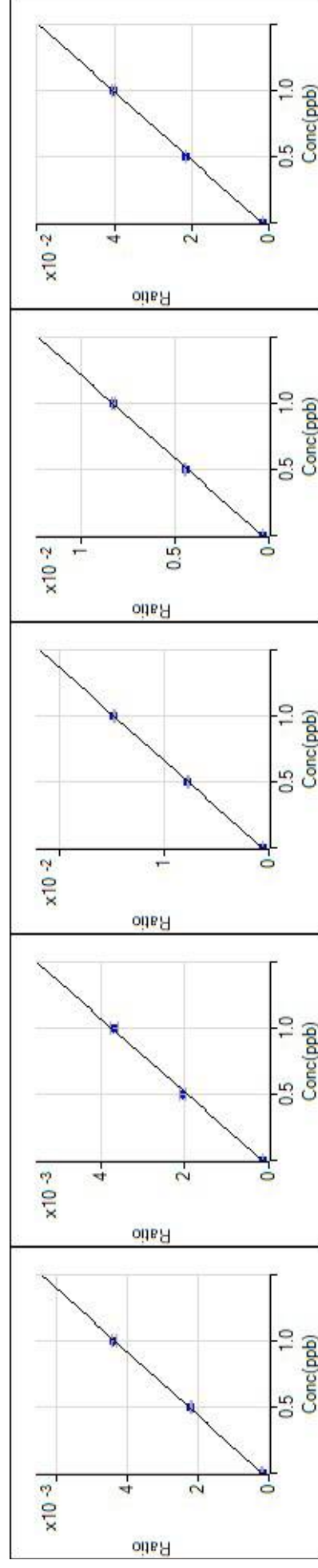

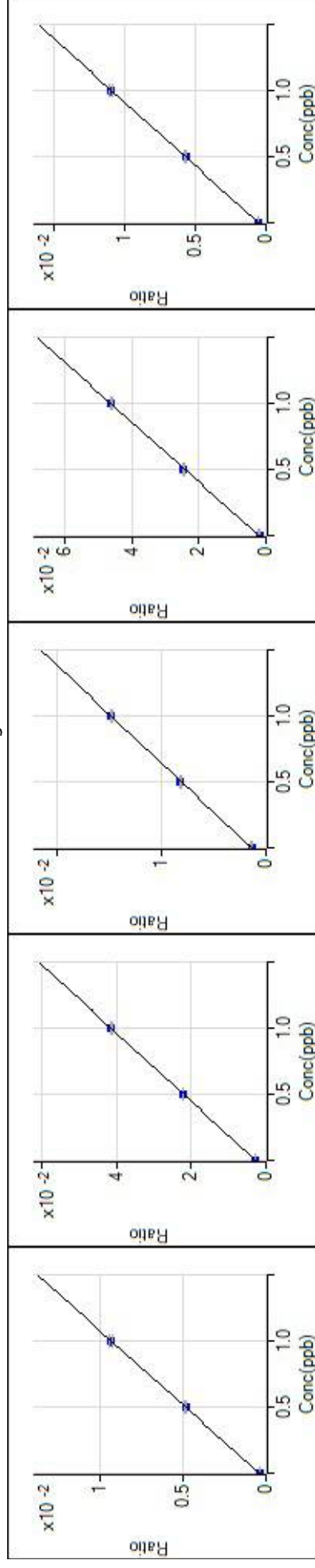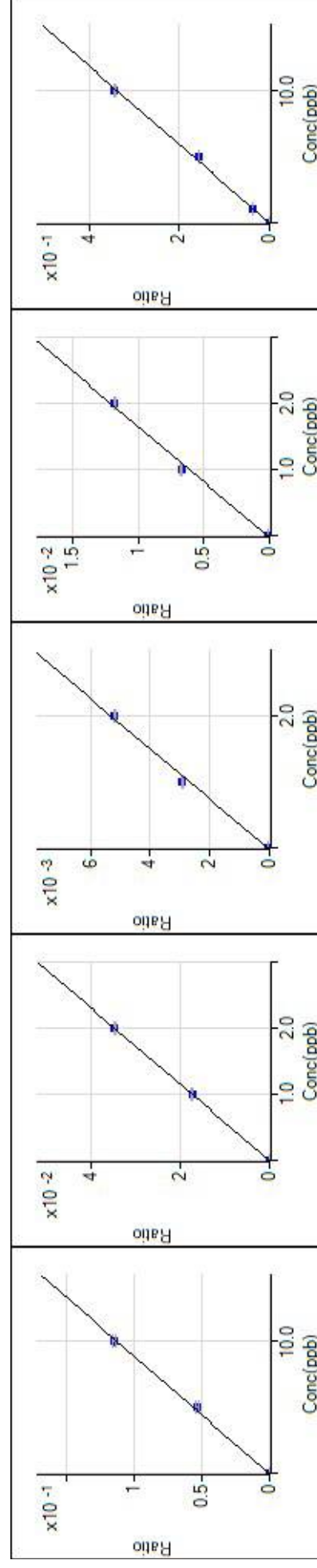

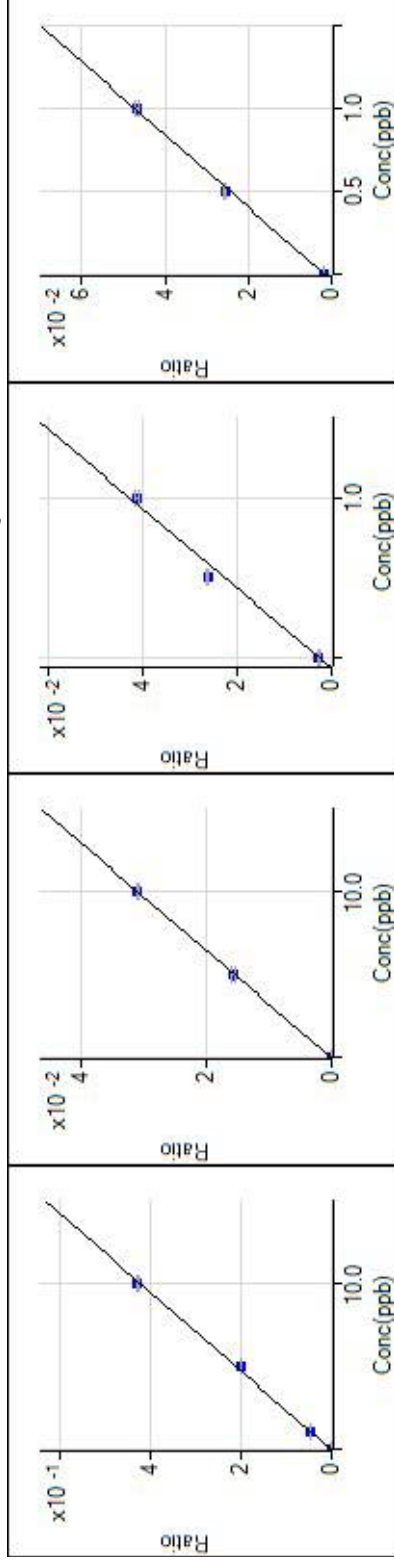

|                             |                             |                             |
|-----------------------------|-----------------------------|-----------------------------|
| 208 Pb [ He ]               | 232 Th [ He ]               | 238 U [ He ]                |
| ISTD: 175 Lu                | ISTD: 175 Lu                | ISTD: 175 Lu                |
| $y = 4.220E-2 x + 1.351E-3$ | $y = 4.023E-2 x + 2.659E-3$ | $y = 4.555E-2 x + 1.674E-3$ |
| R 0.9990                    | R 0.9914                    | R 0.9992                    |
| DL 0.003831                 | DL 0.008847                 | DL 0.004337                 |
| BEC 0.032                   | BEC 0.06611                 | BEC 0.03674                 |

Supplement: Supplementary file 1 [file ijerph-19-04420-s001.zip › ijerph-1642890-supplementary.pdf]
